# Mr. Ring, on Vaccination in Canada

**Published:** 1806-04

**Authors:** John Ring

**Affiliations:** New Street, Hanover Square


### *To the Editors of the Medical and Phyſical Journal.*

Gentlemen,

In the Frankfort Journal for February, 1806, is the following article. "The reports of several committees of vaccination, established in Spain, as well as in the new world; reports which are all accompanied with attestations delivered by the civil and judicial authorities, unanimously confirm the happy result of this salutary practice.—The natural small-pox, which almost every year desolated Mexico and Peru, has lost its malignity in those climates to such a degree, that the number of children who fall victims to that scourge, is reduced in the proportion of nine to one."

On a former occasion I communicated the intelligence, which I received from Dr. Longmore, of Quebec, relative to the practice of vaccination, which he had a second time introduced into that remote corner of the British dominions, by means of armed vaccinators, sent by me.—About a year ago, I received further dispatches from him, dated November 23, 1804, in which he informs me, that although he had at first met with great obstacles, from the extreme ignorance, prejudice and superstition, which prevail among the bulk of the inhabitants of that province, yet he had been able to propagate the practice to a considerable extent.

He had also been so fortunate as to transmit the virus, with instructions, to some of the more intelligent inhabitants of lower Canada. Mr. Fox, a magistrate, of Isle Percée, in the district of Gaspée, on the Gulf of St. Lawrence, has also done much in this great cause of humanity; as appears from his letter, which Dr. Longmore prevailed on him to publish in the Quebec Gazette, in order to stimulate others to the same benevolent exertions. This letter he has transmitted for my perusal.

He has also received similar communications from several other gentlemen, residing along that coast, stating the extent and success of their endeavours; and he is of opinion, that the blessings resulting from vaccination have in no instance been more conspicuous, or more gratefully received, than among the poor inhabitants of this district. Dr. Longmore says, "their situation was peculiarly distressing, when this sovereign antidote was sent for their relief." He says, the small-pox then prevailed there for the the first time, so that men, women, and children, were indiscriminately exposed to the contagion; and being entirely destitute of medical assistance, numbers must otherwise have fallen victims to the ravages of that dreadful disease.

The terror into which they were thrown, overcame every prejudice, and determined them universally to adopt vaccination; which was practised by the most enlightened of them, with complete success. But the advantages of the practice have not been confined to this district: they have also been extended along the shores of the New Brunswick side of the Bay of Chaleurs, in the Gulf of St. Lawrence; and the majority of the inhabitants of those settlements have adopted vaccination.

Dr. Longmore has also transmitted letters to shew, that his exertions, and those of other philanthropists, have not terminated with man in his civilised state. The tribe of Mickmack Indians have also participated in the blessings of this greatest, and most beneficial of all discoveries; particularly to them, for their dread of the small-pox is beyond all description; and to them the disease generally proves fatal. The expressions of gratitude, in the letter of their chief, may therefore be considered as sincere.

The first letter alluded to by Dr. Longmore, was published in the supplement to the Quebec Gazette, in October, 1803, and signed Theophilus Fox, I. P. Isle Percée, district of Gaspée. It there appears, that this worthy magistrate had a very arduous task to perform; a spurious disease having been previously propagated in the district, and many lives having been lost in consequence of a supposed security. At length, however, he surmounted all difficulties by his ardour and perseverance; in which he was ably seconded by the Rev. M. Le François, Curé of Bonaventure. Both these gentlemen acted the humane part of practising vaccination, and putting their patients to the test of variolous inoculation, with their own hands.

Mr. Fox informs us, that there is not a single person within ten leagues of his residence, whom he has not converted; and no one who has not either gone through the small-pox, or vaccine inoculation. Among the latter were several women, far advanced in their pregnancy, and children only a few hours old; none of whom received the least injury from the operation. Having giving an interesting detail of his practice, and of the impression it made on the minds of his patients, he expresses his gratitude to Divine Providence, for enabling him to rescue so many of his his fellow creatures, from the most fatal calamity that ever afflicted mankind.

Mr. Fox concludes his letter with observing, that having done all he could within his reach, he was then preparing vaccinators, with a lancet, to send to the chief of the Mickmack Indians, at Ristigouche. The Rev. M. Le François offered to be the bearer; and having become an expert inoculator, instructed them how to perform the operation. Mr. Fox conceived he could not make a more valuable present to these poor Indians, to whom the small-pox proves so fatal, that it strikes them with a greater terror than the sword. He apologizes for the lengths to which he has gone; and informs us, that vaccination has fixed its reputation among the inhabitants of the district of Gaspée, on the firmest basis, without medical aid.

It appears by a letter from the Rev. M. Le François, to Dr. Longmore, that the Bishop of Quebec, and the Canadian Clergy in general, received the tidings of this happy discovery with a cold indifference, as too many of the clergy and of the laity, in other *civilised* and *enlightened* countries, have done; but it is evident from a letter written by Mr. Edward J. Man, of New Carlisle, to Mr. Fox, transmitted to me, that the unrefined and untutored Indians received it as the gift of heaven.—Mr. Man was desired by Mr. Jacke Gagnion, their chief, in behalf of his tribe, to return Mr. Fox and Dr. Longmore sincere thanks; and to add, that he offered his fervent prayers to the Almighty for their health and welfare; as the only return in his power for so great a blessing.

Almost every member of this tribe had submitted to vaccination, with a perfect confidence in the efficacy of the practice, in which they have not hitherto been disappointed; and, having recommended it in the strongest terms to their brother Indians, throughout Nova Scotia and New Brunswick, no doubt was entertained, that within a few months, it would be generally circulated in that quarter.

I am informed by a gentleman, who lately travelled through the United States, that vaccination is there also making a rapid progress. I long ago stated, that the President, who may so justly be styled the Father of his People, had not only patronized it, but likewise introduced it into his own family, and practised it with his own hand. He also transmitted matter to many of the states. Such an example could not fail to diffuse the blessings of the Jennerian Discovery through that extensive tract of the new world.

I have now before me the Report of the Central Committee of Vaccination at Paris, published in the year 1803. They state, in their preface, that the practice at first met with a powerful opposition in France; as I have mentioned on former occasions, although the contrary has been affirmed in more than one channel. "Chimerical fears were propagated, and imaginary dangers alleged, in order to check its progress. Though the practice was successful, this was not sufficient to prevent its opponents from multiplying those obstacles; and any unfavourable circumstance that appeared, when exaggerated by lying reports, was sufficient to keep the public mind in a state of suspense."—This is an exact counterpart of what has happened in other places; but we are told, that in Paris, the advantages attending the practice were then so manifest, as to silence all opposition.

An address of the Minister of the Interior is prefixed to this work; in which he informs the Committee, that he has read their Report with great interest; and that the Government has seen with pleasure a free and disinterested association zealously occupied in ascertaining, in the most authentic manner, the real advantages of this valuable iscovery. "In the first moments of your re-union," says he, "it has founded on your labours great hopes for the progress of the art, and the consolation of humanity. You have perfectly justified its attention; and your operations, which are crowned with the happiest success, secure you an honourable claim to the gratitude of the public.

"Your Report, and that which has just been made to the National Institute, reflect great light on this salutary practice; and, it appears to me, ought irrevocably to fix the general opinion in its favour. In consequence of this, I invite you, in the name of the public welfare, to continue your experiments; and for this purpose, I will afford you all the assistance of which you have occasion, *by submitting to your disposal a place for vaccination, whenever you desire it; and, if necessary, by appropriating certain funds for the support of the institution.*"

From this and other documents it appears, that although vaccination was discovered in this country, and was not for a considerable time introduced into France, it has there, from its introduction to the present time, met with the countenance and protection of government, which it has never met with here. Fas est et ab hoste doceri.

The French minister approves of the idea of opening a new subscription for the extermination of the small-pox, by by means of vaccination; and says, he shall voluntarily unite in this philanthropic act, requesting that his name may be set down in the list of subscribers for the sum of two thousand francs. What a contrast is this to the conduct of a minister of another country, who was solicited by the Royal Jennerian Society to subscribe to the same philanthropic act, and refused to have his name set down for a single farthing. This was not the minister who held the reins of government, when a remuneration was granted to Dr. Jenner.

The French minister, not content with contributing his pecuniary aid to the promotion of this patriotic design, declares that he intends to excite the emulation and zeal of the committees of vaccination in the departments, and of the different learned societies which are occupied in the practice, that it may triumph over all the obstacles which oppose its adoption.

He also declares, that he will take measures for introducing it into public seminaries and charity schools; and engage the minister at war to extend its benefits to the different establishments within his jurisdiction. He expresses a hope, that the happy results of the new experiments about to be made upon a more extensive scale, would fix the wavering resolutions of those, who still hesitate to acknowledge the advantages of vaccination; and his most ardent wishes, that the fathers of families would imitate the example of government; and hasten the destruction of that scourge, which commits such immense ravages on population.

He tells the committee, that their report cannot be made too public; and desires them to send him five hundred copies, which he may send into all the departments.

Hence it appears, that our enemies eagerly embraced the practice of vaccination, and promoted it with all the just influence and authority of the state; and that their government has set an example which no government on earth should blush to imitate; by exhorting the people to adopt a plan recommended by the most enlighted practioners, not leaving them to be deluded, impoverished, and massacred, by quacks and impostors.

The committee have made the same mistake as their countrymen in general, respecting the first promoters of vaccination in London. They speak of a Doctor Simmons as one of these. This error took its rise from Mr. Simmons, of Manchester, having made experiments with the matter of grease. Those experiments proved unsuccessful; and therefore were far from confirming any one of Dr. Jenner's opinions; but Mr. Simmons has, since that period, adopted vaccination, and united with every other eminent practitioner in England, in recommending the practice.

The committee are decidedly of opinion, both from their own experience and th t of their correspondents, that the cow-pox is not an eruptive disease; and that when vaccine vesicles appear in any other parts, except the puncture, it is in consequence of the insertion of vaccine virus, either by the nails, or some other means. In the last number of the Journal, a contrary opinion is expressed by the reviewer of Mr. Moore's pamphlet, and Dr. Jenner's authority is quoted in favour of that opinion; but the passage of his work, in which he admits this point, is not mentioned. It is, however, contrary to the general sentiment of medical practitioners throughout the world; and some circumstances which have fallen within my own observation, enable me to account for that diversity of opinion. I have known several instances, in which children have been observed to scratch the place of inoculation immediately after the insertion of virus; and to inoculate themselves in other places, particularly in those where they had an eruption; which, by the itching with which it is attended, provokes them to scratch, and by the readiness with which the cuticle is there abraded, facilitates the insertion of matter.

From much experience, numerous inquiries on the subject, and particular attention to this point, I am perfectly convinced that no cow-pock ever appears, but where vaccine virus has been applied. I have known in one instance, five or six rise on the hands and arms, at the same time with the vesicle produced by inoculation with the lancet; but in that case, the child, who was about four years of age, was observed by both its parents to scratch the puncture as soon as she was vaccinated, and then to scratch herself in several other places, where she had been bitten by bugs.

I have also seen, at least a dozen cow-pocks surrounding the original one, but later in their appearance, and smaller. This occurred in a boy about seven years of age, who was almost covered with the itch, but vaccinated before any remedy was employed to cure that disorder, because the small-pox prevailed in the neighbourhood. No instance has occurred within my observation, nor have I been able to hear of any, where a cow-pock appeared in any any place but that of the insertion of virus, in an infant too young to scratch the part affected, and inoculate itself again. In some instances, a second has been occasioned by an accidental puncture of the lancet; and I have known cases, in which this has been noticed by the by-standers, but escaped the observation of the operator himself.

The committee have observed, what has also been repeatedly noticed by others, that the modes of inoculating by means of a blister, or of thread, are more fallacious than others, on account of the suppuration, and spurious pustules thus occasioned. They likewise remark, that among other causes of pustules of this kind, which are doubtful and obscure, there are two principal ones, which are evident from their own experience; first, the insertion of vaccine virus into a subject that has had the small-pox; secondly, the insertion of matter taken from a cow-pock at too late a period.

After adducing the strongest arguments, and the most striking facts, to prove the safety, mildness, and efficacy of vaccination, the committee observe, that in addition to the other attacks made on the character of the cow-pock, its very existence has been denied; and it has been thought by some persons to be only a species of small-pox, mitigated by transmission from the human subject through the brute animal. This hypothesis they consider as visionary, and think it can only arise from want of reflection. In their opinion, it is absurd to suppose, that variolous matter can be sweetened, or rendered milder, by passing through the nipple of a cow.

The committee state, that Cit. Tessier, member of the national institute, had made experiments, in order to ascertain, how far it was possible to transfer the eruptive diseases of animals of one species to those of another. He found it as easy to inoculate sheep with the matter of the sheep-pox, as human subjects with the matter of the small-pox; but did not succeed in his attempts to inoculate other animals with the matter of the sheep-pock. The committee had tried these experiments, but with no better success. A great number of attempts were made eight years before, at the School of Medicine. Variolous matter was taken from pustules still remaining in a body buried more than twenty years. With this, and with recent variolous matter, a great number of animals were inoculated, such as sheep, apes, dogs, rabbits, and birds of different kinds. Matter of the sheep-pock was also tried on several sorts of animals, in order to ascertain their comparative susceptibility, but it only succeeded in sheep.

The committee observe, that Mr. Simmons had also inoculated two cows with small-pox matter, but without effect. Cit. Marchier, of Peronne, also inoculated sheep for the small-pox; in consequence of which, small white oblong pustules appeared on the fifth day, which neither resembled the small-pox, nor the cow-pox. With matter taken from these pustules, on the seventh day, he inoculated a child, but without producing any disease.

The Jury of Health and the Medical Committee of the department of the Somme, inoculated a cow, in the nipple, with matter of the confluent small-pox, by eight punctures, but produced nothing more than an eruption of pimples, of the size of millet seeds, not bearing the least resemblance to the small-pox.

Lastly, Cit. Voisin informed the committee, that he had inoculated a number of cows with small-pox matter, and others with a mixture of small-pock and cow-pock matter; but without effect. The committee, therefore, consider the transmission of variolous matter through the brute animal as a gratuitous supposition; and that, as to the modification of the virus by any such means, it is an idea contrary to all observation and analogy. We have no proof that any morbid poison is mitigated in such a manner. Even the cow-pock itself, among the few diseases communicated to us by the brute animal, is a proof of the contrary; since it is perpetuated in the human subject by successive inoculations, without undergoing any change. These are sentiments which I have often expressed, and which I published long before the report of the committee fell into my hands.

Having given a satisfactory account of their labours, the committee conclude their report with declaring, that all which has been asserted in favour of vaccination is now confirmed; and that they are perfectly convinced of the reality of the advantages ascribed to this new practice. Every thing proves, of what service it would be to all mankind, that it might be universally propagated; and that this plan, which is the offspring of an enlightened humanity, might be easily carried into execution. They also declare, that they cannot conclude their report, without returning a just tribute of acknowledgement to the illustrious author of this discovery, Dr. Jenner; being fully persuaded, that he will hereafter be remembered among among those who have reflected the greatest honour on science, and rendered the most important service to mankind.

The second annual report, lately published by the Cow-pock Institution, in Dublin, patronized by his Excellency the Lord Lieutenant, confirms in all the most essential points, that of the central committee at Paris; giving an additional sanction to the practice of vaccination, and still further refuting the unfavourable reports which have lately been propagated with so much industry in this metropolis.

The physicians of that institution, Drs. Clarke, Cleghorn, and Evory, and the Surgeons, Mr. Stewart, Mr. S. Obre, and Mr. Richards, remark, that this report differs in no essential point from that of last year; that in every instance the cow-pock observed its usual mildness, no symptom occurring to excite anxiety for the safety of the patients, although their number amounted to one thousand and thirty-two. They also declare, that no vaccine vesicles appeared, but what were obviously produced by an accidental application of the virus from the arm.

They remark, the small-pox may appear at any period of the cow-pock, before the formation of the areola. I have, however, repeatedly known it appear during and after the formation of the areola. One case of this kind is under my care at the present time, in a child of Mr. Young, No. 33, Swallow-street, exposed to variolous infection three days before vaccination. In this case the areola began to form on the ninth day, and the small-pox to appear at the same period. The areola has pursued its regular course, and the vesicle is gradually turning into a dark-coloured scab. The small-pox is extremely mild.

They state, that children affected with scrofula, rickets, and other chronic complaints, went through the cow-pock, as if no such diseases were present. On the contrary, Dr. Clarke, of Nottingham, in his letter to Dr. Walker, published in the last number of your Journal, describes a peculiar kind of scab, following a regular vesicle, in persons who are scorbutic. I should esteem it a favour, if Dr. Clarke would inform me, either through the medium of this Journal, or in any other mode, what are the symptoms in such patients, which he deems scorbutic.

In a note on another part of Dr. Clarke's letter, Dr. Walker delivers an opinion, which I consider highly dangerous, gerous, and contrary to that laid down by all the best writers on the subject. It tends to lessen that extreme caution and circumspection, which all the other labourers in the vineyard of vaccination have so earnestly endeavoured to inculcate. He says, "when the pock has been ruptured, and even almost obliterated, if I can feel, about the tenth day, a degree of hardness and tumefaction about the inoculated part, I find, in people of every colour, that the protection is complete." This observation is calculated to put inoculators off their guard; and to render all the instructions of all the vaccine societies, as well as of individuals, on this point, null and void. If, after the rupture of the cow-pock, no vesication re-appears, it has hitherto been universally deemed necessary to repeat the operation; which is attended with very little trouble; and from which no material harm is likely to ensue. As to the induration and tumefaction, subsequent to the rupture of a vesicle, they are, in some measure, the necessary consequence of that accident; partly from the injury itself, and partly from the irritation of the air, and perhaps of the nails, as well as the clothes of the patient. I had, indeed, seen one instance, in which a child was pronounced safe on the fifth day, and brought to me on the sixth, by desire of an eminent practitioner in midwifery. In this child, there were two light brown scabs, but no remaining vesication. I requested the mother, who lived at a distance, to bring the child again, that I might watch the progress of the case, and re-inoculate it, but I saw her no more. The mention of such a case, however, will not be useless, if it serves to put the inoculator in question, a little more on his guard in future.

I could not quit this subject, without entering my protest against such dangerous doctrines. The light brown scab is no test of security; the induration or tumefaction is no test of security. *If no case can be secure without induration or tumefaction,* it by no means follows that the converse of this proposition is true; and that every case is secure, when either induration or tumefaction occurs.

I cannot, at this moment, recollect any place, where mention is made of the suspension of the cow-pock, and its continuing stationary, in consequence of other diseases under which the patient happens to labour at the same time, as noticed by Dr. Clarke; but it is a very common occurrence, and probably would be more alluded to in treatises on this subject, did it not agree with our experience

-ence in other disorders. It is universally admitted, that one morbid action *generally* suspends another; it is therefore the exception that has commonly been thought worthy of notice, and not the rule. I have known a vaccine vesicle, when arrived at the eighth day, continue nearly stationary above a fortnight, and yield very active matter at last.

This circumstance of the exsiccation not proceeding, while the vital powers are weakened, convinces me that absorption is more or less suspended during that time; and the activity of the dry matter remaining in the scab, when dissolved, proves that little more than the aqueous portion of it is absorbed at last.

This document contains the testimony of the very respectable medical practitioners who superintend the Vaccine Institution in Dublin, whose names are mentioned above, that no diseases have been observed to follow vaccination, which could be imputed to that cause. They conclude their very valuable Report in these words. "No case occurred to excite doubt, as to the permanent efficacy of cow-pock in protecting the system against the small-pox, either in this Institution, or in the private practice of any of the gentlemen who superintend it; although a most malignant small-pox raged very generally, throughout this city and its neighbourhood, during the last summer.

"Since applications for cow-pock matter, and for inoculation, continue to increase daily, it is hoped that the period is not distant, when its salutary influence shall be as generally experienced in Ireland, as in Great Britain."

This Report, having been transmitted to the Vaccine Pock Institution, the medical officers of that establishment declare, that their experience agrees with that of the institution in Dublin, with regard to the uniform mildness of the cow-pock; no dangerous symptoms having appeared, unless the patient laboured under some other disorder at the same time. They agree with the gentlemen of the Vaccine Institution in Dublin, and with the opinion I have repeatedly expressed, that whenever vaccine vesicles have appeared in other parts, they have been produced by the application of vaccine matter to such parts. But they observe, that they do not here speak of those pimples, or small eruptions, like the tooth-rash, which often occur between the tenth and twentieth day after vaccination; and seem to depend on the constitutional disorder.

The medical officers of the Vaccine Pock Institution agree with the author of these remarks, that herpetic eruptions, eruptions, as far as they have seen, have seldom occasioned any difficulty in communicating the infection of the cow-pock, nor altered its course, except from the patients, in such cases, scratching or rubbing their arms; and that, in general, neither scrofula, the rickets, nor any other chronic complaint, has interrupted the regular progress of this affection.

They admit, that they have had one case of the small-pox after vaccination, as far as they can trust their register; but they tell us, that whether the patient, in this instance, had really had the cow-pox, must be left to the determination *of others*. For my own part, I leave it to the determination of one of themselves, Dr. Nelson, the very person to whom the case belonged; who positively assures me, that the patient regularly went through the cow-pock, under his own inspection. The subject was a child of Mr. M'Pherson in Fitzroy Market; and I am one of the many witnesses, who saw sufficient proofs that it was followed by the small-pox.

I am, &c.

JOHN RING.

*New Street, Hanover Square.*

